# Evaluation of Vulnerability Factors for Developing Stress and Depression due to COVID-19 Spread and its Associated Lockdown

**DOI:** 10.2174/17450179-v18-e2209291

**Published:** 2022-10-06

**Authors:** Ahmed Alhusban, Karem H. Alzoubi, Sayer Al-Azzam, Khawla Q Nuseir

**Affiliations:** 1 Department of Pharmacy Practice and Pharmacotherapeutics, College of Pharmacy, University of Sharjah, Sharjah, United Arab Emirates; 2 Department of Clinical Pharmacy, Faculty of Pharmacy, Jordan University of Science and Technology, Irbid, Jordan

**Keywords:** Coronavirus, COVID-19, Mental health, Depression, Stress, Public

## Abstract

**Background:**

COVID-19 is a pandemic that has been widespread throughout the world. The disease and the measures employed to contain its spread have a detrimental effect on the mental health of individuals. Countries across the world have applied variable combinations of quarantine and social distancing measures to contain the spread of COVID-19. This project aims at identifying the susceptible groups for the development of depression and stress due to COVID-19-associated containment measures. This evaluation will help in prioritizing efforts to ameliorate the detrimental effects of COVID-19 on psychological health.

**Methods:**

A cross-sectional study was conducted through an online survey that included questions on the demographics and COVID-19 experience. The prevalence of depressive symptoms was evaluated using the PHQ-9 survey, whereas stress levels were detected using the perceived stress scale (PSS). Data regarding demographics as well as exposure to COVID-19, working at home and the financial impact of the pandemic were collected.

**Results:**

Data were collected from 1541 participants from the MENA region. Depressive symptoms were detected in 54.2% of the participants, and the average stress score was 18.4±0.8. Adjusting for demographics and other variables, younger participants were more likely to report depressive symptoms and higher stress scores. Additionally, younger age, female gender, the coexistence of depressive symptoms, negative effects on monthly income, and ability to do work were found to be independent predictors of higher stress scores.

**Conclusion:**

Young individuals are more likely to develop depression symptoms and stress. Thus, there is a need for prompt measures to alleviate COVID-19-associated effects on this group.

## 1 INTRODUCTION

COVID-19 is a pandemic that has spread throughout the world [1]. This pandemic has affected more than 500 million individuals and resulted in more than six million deaths worldwide. The first reported case in the Middle East and North Africa (MENA) region was in the last week of January 2020 [2]. Since then, the pandemic has spread all over the region with variable number of cases and mortality [1].

In response to the pandemic, many countries around the world have imposed restrictions on individuals’ mobility and several normal daily life activities, including schools, which have led to the introduction of new paradigms, such as distance learning [1, 3-12]. These essential measures have helped in limiting the spread of the disease [5, 13-18] but severely affected the mental health as well as the quality of life of the individuals affected by these measures [3, 4, 19-23]. Following the suspension of these measures, many countries have witnessed an increase in the incidence of cases, and some of these measures have been reinstated to variable degrees [24-27]. Furthermore, the spread of COVID-19 continued to affect various aspects of daily life and created a new pattern of human living and interactions [11, 28-31]. Additionally, this pandemic has created many challenges that are not limited to health and economy. A significant but overlooked challenge at this phase of the international response to COVID-19 is the psychological health and the long-term consequences of psychiatric ailments [3, 7, 12, 23, 32-35]. Accordingly, identifying vulnerable groups and promoting proactive designs of group-specific interventions are an essential aspect to ameliorate the detrimental COVID-19-induced effects on psychological health.

In this investigation, we aimed to identify the characteristics of the vulnerable groups in a highly dynamic population across the Middle East and North Africa Region (MENA). This evaluation is an initiative to launch a coordinated proactive response to mitigate the long-term effects of COVID-19 on the health, well-being, and productivity of humans.

## 2 MATERIALS AND METHODS

### 2.1 Design

An online survey was designed that included forty questions with an estimated 6 minutes to be completed. A brief explanation of the study, data protection assurance, and consent were included. The study was approved by the institutional review board at the Jordan University of Science and Technology, Irbid-Jordan (approval number: 318/2020). The survey was conducted from May 14^th^ to 17^th^, 2020.

### 2.2 Participants

Participants were recruited through an online invitation distributed through different social media applications. Using the snowball method, the final sample included 1541 participants from different countries in the MENA region.

### 2.3 Variables and Instruments

The following variables and instruments were used in this study: sociodemographic variables and data regarding COVID-19 exposure and experience were collected. Impact on mental health was assessed using the Arabic versions of the Patient Health Questionnaire-9 (PHQ-9) [36] and the Perceived Stress Scale (PSS) [37]. Both tools are previously validated brief self-reported questionnaires that assess the presence of depressive and stress symptoms, respectively [36, 37].

The PHQ-9 is a self-reported version of the PRIME-MD diagnostic tool [38]. It consists of 9 items measured on a 4-points Likert scale ranging from “not at all: to “nearly every day”. The maximum score in this tool is twenty-seven, and a cutoff point of more than 9 has been suggested to identify patients with depression [6]. The tool was translated into Arabic language and validated in different geographical parts of the Middle East and North Africa (MENA). Becker *et al*. demonstrated the utility of PHQ-9 as a valid instrument to screen for depression in primary settings [39]. Similarly, studies from Saudi Arabia, Lebanon and Tunisia demonstrated the reliability and validity of the Arabic version of PHQ-9 to detect depression [36, 39-42].

The PSS is a self-reported questionnaire that is based on the psychologic conceptualization of stress [43]. It assesses the extent to which life events have been perceived as uncontrollable and unpredictable over the last month [43]. The PSS consists of 10 items on a 5-point Likert scale ranging from “Never” to “Very often”. The maximum score of this tool is 40, with higher values indicating higher levels of stress [43].

### 2.4 Analysis

Descriptive data were calculated for the sociodemographic variables. The associations of the target symptoms to the sociodemographic variables were examined using the χ^2^ test, the unpaired t-test, or one-way analysis of variance (ANOVA), followed by Bonferroni post hoc analysis as appropriate. Odds ratio and the corresponding 95% confidence intervals were calculated for the presence of depressive symptoms using a stepwise regression analysis model. A linear regression model was calculated for the extent of stress symptoms using a forward regression approach. All statistical analyses were performed using the statistical package for the social sciences (SPSS) version 24 (IBM SPSS Statistics for Windows, Version 24.0. Armonk, NY: IBM Corp.). A *p*-value of less than 0.05 was considered statistically significant.

## 3 RESULTS

### 3.1 Participants’ Demographics

In this study, we recruited 1541 participants with an average age of 31.9 years (SD=0.8). More than 80% of the participants were younger than 40 years. The study included 960 female participants (62.3%), and about half of the participants had a bachelor’s degree (761, 49.4%). Exposure to COVID-19 patients was reported by 91 participants (5.9%). Complete lockdown was reported by 567 (36.8%) of the participants, and 820 (53.2%) reported working from home. A length of more than 8 weeks of lockdown was reported by 942 (61.1%) participants and 772 (50.1%) participants reported a negative effect on their monthly outcome as a result of the pandemic. Table **1** provides a detailed description of the demographic data of the participants.

### 3.2 Psychological Target Symptoms

Depressive symptoms exceeding the cutoff point were detected in 835 (54.2%) participants with an average of 11.2 (SD=7.02). The average score of the PSS was 18.52 (SD=4.47), with more than 86.6% experiencing moderate to high levels of stress (Score > 14).

### 3.3 The Distribution of Target Symptoms Stratified According to Participant’s Demographics

Depression was detected in more than half of participants under the age of 40. More than 75% of the participants in the 18-20 years age group were found to have depression (204 participants). Younger individuals were more likely to have depression (χ^2^ =80.2; p<0.0001). Similarly, younger individuals (18-20 years group) had significantly higher levels of stress (mean 20.69; p<0.0001).

Depression was less likely to be reported among participants working for the government or the private sector (χ2 =39; p<0.0001) compared to self-employed and unemployed. Similarly, those working in government or for private sector had lower stress scores compared to self-employed and unemployed. Lockdown for more than 8 weeks was associated with higher levels of stress (18.82; p=0.016) and depression symptoms (543; 57.6%; χ^2^= 11, p=0.01).

Participants with known exposure status to COVID-19, whether exposed (91 participants) or not (1277 participants), had a similar extent of depression (50.5% *vs*. 53%). On the other hand, participants with unknown exposure status were more likely to have depression symptoms (64.9%; χ^2^=9.9, p=0.01). Table **2** provides a detailed description of the distribution of target symptoms stratified according to the demographic variables.

### 3.4 Predictors of Depression and Stress

Multivariate logistic regression analysis identified age, country of residence, school attendance, effect on monthly income, working from home, and working performance as independent predictors of developing depression. High school students (OR=1.89; 95% CI:1.01-3.56) and undergraduate students (OR=1.861; 95% CI:1.31-2.65) had increased risk for developing depression. Furthermore, participants who were unsure whether their monthly income will be negatively affected had a one-fold increase in the risk of developing depression (OR=1.8; 95% CI: 1.27-2.56), whereas those who had their monthly income decreased showed a 0.5-fold increase in the risk of developing depression symptoms (OR=1.5; 95% CI: 1.16-1.98). Table **3** provides the details of the multivariate logistic regression model.

The linear regression model of stress symptoms explained the variance by 38.3% (F (6,1497) =155.49; p<0.0001). Factors, such as age category, concomitant depression, and country of residence, were identified as significant variables of stress. Table **4** provides the details of the linear regression model.

## 4 DISCUSSION

In this paper, we have reported an assessment of vulnerable groups for the psychological health detrimental impact of COVID-19. Current data suggest a dramatic effect of COVID-19 on the mental health of the population of the MENA region. The overall prevalence of depression in the current sample was about 50%. Similarly, the average stress score was 18.4. Current results identified age, country of residence, and the effect on monthly income and work performance as common predictors for both stress and depression.

Depression and stress are frequently encountered at times of distress [4, 6, 19, 33, 34]. The prevalence of depressive symptoms in the current sample was about 50%. This high value has exceeded the reported levels of depression symptoms in other parts of the world during this epidemic [4, 6, 7, 32-34]. A recent meta-analysis that evaluated the effect of COVID-19 on the mental health among health care providers reported a pooled 20% prevalence of depression symptoms [33]. Similarly, González-Sanguino *et al*. reported a 19% prevalence of depression symptoms among participants from Spain [4]. Furthermore, Carmassi *et al*. reported the development of depression and PTSD among health care providers in the epicenter of COVID-19 in Italy [35]. Additionally, a public-based study in China reported depression symptoms in less than 20% of the evaluated sample during the initial phases of the pandemic [44]. On the other hand, Gao *et al*. reported depression symptoms in about half of the sample that included more than 4000 participants from China [21]. The variability in the prevalence of depression symptoms might be related to the timing of the assessment. Studies conducted early during the pandemic showed lower levels of depression symptoms [4, 32, 44, 45], whereas assessments made one month after the pandemic showed higher levels of depression symptoms [21]. This explanation is further supported by data from the current study, which showed that participants who experienced longer durations of lockdown had the highest prevalence of depression symptoms. Similarly, symptoms of moderate to severe stress were reported by more than 80% of the participants. The high prevalence of stress symptoms exceeds the levels reported in studies during this pandemic [3, 4, 19, 21, 32-34].

The prevalence of both depression and stress is affected by a number of common factors, such as age and country of residence, work performance, and the effect of the pandemic on monthly income. Interestingly, current data show that younger individuals are more likely to report symptoms of depression and stress. These findings are consistent with other reports from different parts of the world [4, 7, 21, 32, 34, 44]. These findings should be carefully evaluated among elderly patients who have been consistently underrepresented in the reported studies [4, 21]. Furthermore, the effect of the pandemic on monthly income has been identified as a predictor of both depression and stress. This can be explained by the lockdown measures employed in many countries and the inability to work and generate income during this period. Support for this explanation is provided by the finding that self-employed or unemployed participants are more likely to report depression and stress symptoms.

This study has a number of limitations, such as the variability in response rates from different countries in the region. The majority of the participants were from Jordan and Algeria, with lower numbers from UAE, Egypt, and KSA. Additionally, the online nature of the survey may have introduced a bias toward younger ages, individuals with more literacy and higher educational levels. Furthermore, the short duration of the study may have affected the representation of various parts of the MENA region. This study employed a snowball approach to sampling, which has been used in similar studies during the same period [7, 21, 34, 44, 46]. This approach is a convenience sampling method that has many limitations, but it is a pragmatic method under the pandemic situation. Furthermore, this method makes it challenging to track the nonresponse rate, which is very important to better understand the study population and the results. Finally, there seems to be a possibility of an interaction between stress and depression, which was investigated using the linear regression model for stress. Depression is a significant predictor of anxiety, according to the analysis. The possibility of having severe stress that is misidentified as depression is valid. To account for that, we reanalyzed the data and found that the sample included 69 (4.5%) subjects with severe stress along with depression. Accordingly, this possibility of interaction between stress and depression needs further future studies.

## CONCLUSION

COVID-19 has exerted a dramatic effect on mental health in the MENA region and should be evaluated in more depth to assess its causes and long-term effects. Special attention should be given to the more vulnerable groups, such as younger individuals and people with unstable income.

## Figures and Tables

**Table 1:** Baseline characteristics of the participants.

Variables	
**Age (y), Mean± SD**	31.9±50.8
**Age Categories, n (%)**	-
18-20	626 (40.6)
21-40	660 (42.9)
41-60	238 (15.3)
>60	17 (1.1)
**Gender, n (%)**	-
Male	581 (37.7)
Female	960 (62.3)
**Marital Status, n (%)**	-
Single	801 (52)
Married	701 (45.5)
Divorced	39 (2.5)
**Country of Residence, n (%)**	-
Jordan	467 (30.3)
United Arab Emirates	209 (13.6)
Saudi Arabia	94 (6.1)
Algeria	448 (29.1)
Egypt	202 (13.1)
Other	121 (7.9)
**Employment, n (%)**	-
Government	509 (33.4)
Private	371 (24.3)
Self employed	174 (11.3)
Unemployed (including students)	487 (31.6)
**Educational Level**	-
Less than high school	110 (7.1)
High school	361 (23.4)
Bachelor	761 (49.4)
Master’s degree	231 (15)
Ph.D.	78 (5.1)
**School Attendance, n (%)**	-
Not a student	982 (63.7)
High school	136 (8.8)
Undergraduate	301 (19.6)
Graduate	122 (7.9)
**Occupation, n (%)**	-
Medical	471 (30.6)
Non-medical	1070 (69.4)
**Contact with COVID-19 Patients, n (%)**	-
Yes	91 (5.9)
No	1277 (82.9)
Not sure	173 (11.2)
**Lockdown Type, n (%)**	-
Complete	567 (36.8)
Partial	909 (59)
None	65 (4.3)
**Duration of Lockdown, n (%)**	-
< 1 week	120 (7.8)
2-3 weeks	128 (8.3)
4-8 weeks	351 (22.8)
>8 weeks	942 (61.1)
**Monthly Income Negatively Affected, n (%)**	-
Yes	772 (50.1)
No	514 (33.4)
Not sure	255 (16.5)
**Worked from Home, n (%)**	-
Yes	820 (53.2)
No	721 (46.8)
**Work Performance, n (%)**	-
Negative	878 (57.2)
Positive	127 (8.2)
No effect	536 (34.8)

**Table 2:** Target symptoms stratified according to participants' baseline characteristics.

Variable	**Depressive Symptoms**	**Χ^2^**	**p-value**	**Average Stress Score**	**p-value**
**Total, n (%)**	835 (54.2)	-	-	18.4	-
**Age Categories, n (%)**	-	80.2	<0.0001	-	<0.0001
18-20	204 (75.6)	-	-	20.69^a^	-
21-40	537 (52.6)	-	-	18.37^b^	-
41-60	91 (37.4)	-	-	16.88^b^	-
>60	3 (0.3)	-	-	15.00^b^	-
**Gender, n (%)**	-	-	-	-	-
Male	303 (52.2)	1.5	0.212	17.84	<0.0001
Female	532 (55.4)	-	-	18.93	-
**Marital Status, n (%)**	-	57.4	<0.0001	-	<0.0001
Single	125 (44.1)	-	-	19.3^a, c^	-
Married	76 (63.4)	-	-	17.6^b^	-
Divorced	5 (46.2)	-	-	18.1^b, c^	-
**Country of Residence, n (%)**	-	63.46	<0.0001	-	<0.0001
Jordan	227 (49)	-	-	17.88^a^	-
United Arab Emirates	100 (48)	-	-	18.66^a^	-
Saudi Arabia	39 (41)	-	-	16.87^a^	-
Algeria	254 (56.6)	-	-	18.38^a^	-
Egypt	157 (77.7)	-	-	21.09^b^	-
Other	58 (47.9)	-	-	18.30^a^	-
**Employment, n (%)**	-	39	<0.0001	-	<0.0001
Government	242 (47.5)	-	-	17.59^a^	-
Private	178 (47.5)	-	-	18.20^a^	-
Self employed	259 (64.1)	-	-	19.47^b^	-
Unemployed (including students)	147 (65.0)	-	-	19.21^b^	-
**Educational Level**	-	33.3	<0.0001	-	<0.0001
Less than high school	71 (53.5)	-	-	18.41^a, c^	-
High school	224 (46.6)	-	-	17.84^a, c^	-
Bachelor	407 (33.3)	-	-	16.64^b, c^	-
Master’s degree	107 (64.5)	-	-	19.64^a, d^	-
Ph.D.	26 (62.0)	-	-	19.26^b, d^	-
**School Attendance, n (%)**	-	79.48	<0.0001	-	-
Not a student	458 (46.7)	-	-	17.86^a^	-
High school	105 (77.2)	-	-	20.74^b^	-
Undergraduate	209 (69.4)	-	-	19.75^a^	-
Graduate	63 (51.6)	-	-	18.48^a^	-
**Occupation, n (%)**	-	2.7	0.1	-	0.3
Medical	242 (51.4)	-	-	18.38	-
Non-medical	590 (55.9)	-	-	18.62	-
**Contact with COVID-19 Patients**	-	9.1	0.01	-	-
Yes	46 (50.5)	-	-	18.54^a^	-
No	677 (53.0)	-	-	18.37^a^	-
Not sure	111 (64.9)	-	-	19.65^b^	-
**Lockdown Type**	-	3.9	0.1	-	0.11
Complete	325 (57.3)	-	-	18.84^a^	-
Partial	479 (52.7)	-	-	18.34^a^	-
None	31 (48.4)	-	-	18.30^a^	-
**Duration of Lockdown**	-	11	0.01	-	0.016
< 1 week	57 (48.7)	-	-	18.21^a^	-
2-3 weeks	63 (49.2)	-	-	18.08^a^	-
4-8 weeks	172 (49)	-	-	18.02^a^	-
>8 weeks	543 (57.6)	-	-	18.82^b^	-
**Monthly Income Negatively Affected, n (%)**	-	34.72	<0.0001	-	<0.0001
Yes	454 (58.8)	-	-	19.04^a^	-
No	225 (43.8)	-	-	17.80^b^	-
Not sure	153 (61.7)	-	-	18.44^a^	-
**Worked From Home, n (%)**	-	7.13	0.008	-	<0.0001
Yes	468 (57.4)	-	-	18.92	-
No	365 (50.6)	-	-	18.07	-
**Work Performance, n (%)**	-	189.38	<0.0001	-	<0.0001
Negative	609 (69.3)	-	-	19.88^a^	-
Positive	50 (39.4)	-	-	16.65^b^	-
No effect	174 (32.9)	-	-	16.74^b^	-

Note: Pairs identified with different letters are significantly different from each other

**Table 3:** Multivariate logistic regression analysis of depressive symptoms.

**Variable**	OR (95% CI)	p-value
**Age Categories**		
18-20	1	-
21-40	0.593 (0.37-0.95)	0.029
41-60	0.410 (0.24-0.72)	0.002
>60	0.333 (0.06-1.84)	0.207
**Country of Residence**		
Jordan	1	-
United Arab Emirates	1.18 (0.81-1.71)	0.385
Saudi Arabia	0.98 (0.60-1.62)	0.940
Algeria	1.57 (1.16-2.12)	0.004
Egypt	2.65(1.74-4.05)	0.000
Other	0.91 (0.57-1.44)	0.688
**School Attendance**		
Not a student	1	-
High school	1.89 (1.01-3.56)	0.046
Bachelor	1.861 (1.31-2.65)	0.001
Graduate	1.023 (.67-1.57)	0.919
**Monthly Income Negatively Affected**		
No	1	-
Yes	1.517 (1.16-1.98)	0.002
Not sure	1.800 (1.27-2.56)	0.001
**Worked From Home**		
No	1	-
Yes	1.53 (1.20-1.96)	0.001
**Work Performance**		
No effect	1	-
Negative	4.12 (3.19-5.31)	<.0001
Positive	1.31 (0.85-2.03)	0.218

**Table 4:** Linear regression model for perceived stress symptoms.

Variable	B (std)	p-value
Depression	.485^a^	<0.0001
Work performance	-.137^b^	<0.0001
Age category	-.119^c^	<0.0001
Gender	.115^d^	<0.0001
Country of residence	.085^e^	<0.0001
Monthly income negatively affected	.067^f^	0.001

Notes: Adjusted R square: 0.383.

F (6,1497) =155.49

p-value=<0.0001

Reference categories: a=no depression; b=negative effect; c=18-20 years age group; d=male; e=Jordan; f=yes

## Data Availability

The authors confirm that the data supporting the findings of this study are available within the manuscript.

## LIST OF ABBREVIATIONS

PSS: Perceived Stress Scale
COVID-19: Coronavirus-19
MENA: Middle East and North Africa
PHQ-9: Patient Health Questionnaire-9
ANOVA: Analysis of Variance
SPSS: Statistical Package for Social Sciences
